# Financial Stressors and Resources Associated With Financial Exploitation

**DOI:** 10.1093/geroni/igac010

**Published:** 2022-02-28

**Authors:** LaToya Hall, Juno Moray, Evan Gross, Peter A Lichtenberg

**Affiliations:** 1 Institute of Gerontology, Wayne State University, Detroit, Michigan, USA; 2 Department of Psychology, Wayne State University, Detroit, Michigan, USA

**Keywords:** Financial literacy, Financial resources, Financial self-efficacy, Financial stress, Older adults

## Abstract

**Background and Objectives:**

The prevalence of older adult financial exploitation (FE) is increasing. Population-based survey estimates of FE in the older adult population range from 5% to 11%. Given the growing prevalence of FE victimization in older adult populations, understanding the population’s vulnerability to FE has increased in importance. This study investigates a conceptual framework in an attempt to understand how financial stressors and resources are associated with substantiated FE in a sample consisting largely of Black older adults.

**Research Design and Methods:**

The study uses a cross-sectional design to investigate group differences among a total sample of 142 community-dwelling older adult participants, 62 of whom sought services to address FE and 80 with no history of FE.

**Results:**

The group of older adults who sought services to address FE was more likely to be unmarried and had fewer years of education. Measures of financial literacy and perceived financial vulnerability had protective and risk effects, respectively.

**Discussion and Implications:**

The present study found that sociodemographic and financial stress and resource measures have significant relationships with FE. These findings support the conceptual framework describing their relationship. This new conceptual framework provides a guiding factor in better understanding vulnerability to FE in older adults. The study also adds to the paucity of research completed on FE with Black older adults.

**Translational Significance:** This article provides a conceptual model to assist in the understanding of the relationship of financial stressors and resources with the financial exploitation of older adults. The results of the study indicate the importance of assessing financial literacy and perceived financial vulnerability as part of social determinants of health.

Financial exploitation (FE) is defined as the “illegal or improper use of a vulnerable adult’s funds or property for another person’s profit or advantage” (Conrad et al., 2010, p. 758). The study of older adults’ susceptibility to FE is becoming increasingly important due to the growing nature of this issue in society. Population-based surveys have shown that estimates of FE in the older adult population range from 5% to 11% (Acierno et al., 2010; Beach et al., 2016; Laumann et al., 2008; Lichtenberg et al., 2016). Acierno et al. (2010) reported 5.2% of all older adults in their sample had experienced FE the previous year. Laumann et al. (2008) reported 3.5% of their sample had been victims of FE during the previous year. Beach et al. (2010) found 3.5% of their sample reported experiencing FE at least 6 months prior to the interview and almost 10% at some point since turning 60. Furthermore, Lichtenberg et al. (2016) found the prevalence of fraud reports from older adults increased from 5% to 6.1% in just 4 years. In addition, the Consumer Financial Protection Bureau (2019), Suspicious Activity Reports from deposit institutions and financial services businesses increased fourfold in a period of 4 years. While reports did not involve only older adults, around 70% of the reports were made for individuals older than 60, and 33% were for those older than 80. These data may still fail to capture the scope of this problem. It is difficult to know the true prevalence at which older adults experience FE because only one in 25 cases of elder financial abuse is reported to authorities (Storey, 2020). If left unaddressed, FE can potentially become a societal problem of epic proportions, as the number of Americans older than 65 is expected to double in the next 40 years (United States Census Bureau, 2014).

People who experience FE often suffer financial hardship as a result. Until now, FE has been explored as a unitary construct without paying attention to important subgroups among those who experience FE. We argue those who experience FE and financial hardship from that FE represent a particularly important group to identify and study. Given the growing prevalence of FE victimization in older adult populations, understanding the population’s vulnerability to FE causing financial hardship has increased in importance. FE causing financial hardship has been associated with many adverse correlates and outcomes in the older adult population. Poor mental, physical, and cognitive health have all been associated with financial hardship resulting from FE (Hall et al., 2021; Lichtenberg et al., 2019). Research on the associations between financial attitudes, stressors, skills, and FE is largely missing from previous work. This study evaluated a new conceptual framework in an attempt to understand how financial stressors, knowledge, and vulnerabilities are associated with substantiated FE that has led to financial hardship.

## Literature Review

**Figure 1.**
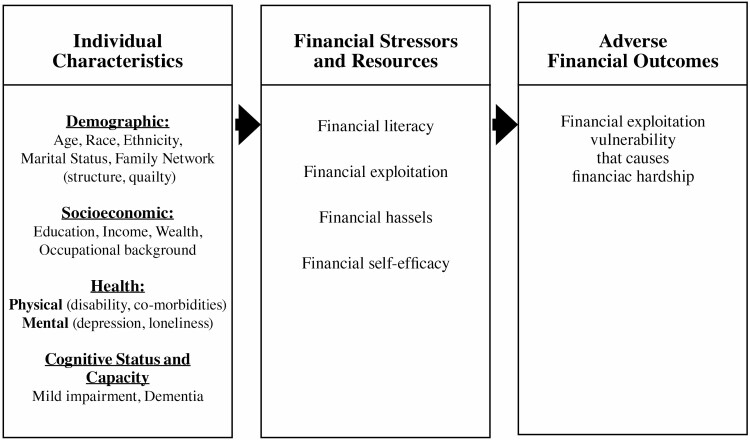
Financial exploitation analytic conceptual framework for older adults.

The proposed conceptual framework is shown in Figure 1 and consists of individual background characteristics, individual financial stressors and resources, and FE causing financial hardship. Individual background characteristics in this proposed conceptual framework include sociodemographic variables as well as physical, cognitive, and mental health variables. Individuals’ financial stressors and resources include the areas of financial literacy (objective and subjective), financial stressors, financial self-efficacy, and FE vulnerability. In the proposed conceptual framework, the background characteristics and the financial stressors and resources combine to predict FE causing financial hardship. Due to the smaller sample size, this study focuses on the sociodemographic and financial measures and their associations with FE that causes financial hardship. The different aspects of financial stressors and resources are examined below.

### Financial Literacy

Financial literacy is defined as the “ability to process economic information and make informed decisions about financial planning, wealth accumulation, debt, and pensions” (Lusardi & Mitchell, 2014). The construct has been commonly operationalized as performance on a financial knowledge assessment or subjective ratings of financial knowledge (Lusardi & Mitchell, 2011). While the content of financial literacy measures varies somewhat, questions typically cover compound interest, inflation, and knowledge about financial investment risk. Lusardi and Mitchell (2008, 2011) reported in previous research that many older adults have trouble understanding basic financial concepts, which indicates low levels of financial literacy. These studies found that many individuals in a community-dwelling sample of older adults lacked the ability to do simple interest rate calculations and did not understand the basic concepts of inflation and risk diversification. However, while age-related financial literacy declines have been noted, older adults do not lose any confidence in their ability to manage finances and make financial decisions. While objective measures of financial literacy declined overall with age, self-assessment of literacy increased with age. The percentage of individuals with high confidence in decision-making abilities (while having low literacy scores on objective items) increased by 20% from age 60 to 85 (Finke et al., 2017). Low levels of financial literacy have also been associated with being more susceptible to being scammed. In a sample of community-dwelling older adults without dementia, financial literacy was negatively associated with being susceptible to scams (James et al., 2014).

### Financial Stress

Marshall et al. (2021) used Health and Retirement Study (HRS) data to investigate financial stress among a sample of adults older than 50. Twenty-one percent of the sample reported persistent financial hardship over an extended period of time. However, more than half of all older adults participating experienced financial hardship at some point since turning 50. An association between financial stress and mental health was also identified. A study conducted on an international sample of older adults reported higher levels of depression and lower self-rated health were related to high levels of adverse financial stress when compared to those with lower levels (Huang et al., 2020). Wilkinson (2016) found financial strain was a strong and robust predictor of worsening mental health when using a subset from the HRS. Examining stress from a daily perspective led researchers to adopt measures of daily financial hassles as a measure of stress. Jacob et al. (2014) adapted a daily hassles measure for low-income populations. Burnett et al. (2020) reported that financial hassles, such as the inability to afford housing, medications, food, and medical care, were predictors of FE.

### Financial Self-Efficacy

Self-efficacy is a measure of a person’s perceived capability and their sense of personal agency. It is an individual’s belief that they can achieve a certain task. Self-efficacy is related to self-confidence, motivation, and optimism (Bandura, 2006). Financial self-efficacy is the measure of self-efficacy as it relates to financial behavior (Lown, 2011). Therefore, an individual’s level of financial self-efficacy is defined as one’s belief in their ability to organize and manage their finances and produce desired results. Financial self-efficacy is related to confidence, motivation, and optimism in financial management abilities.

High financial self-efficacy has been associated with higher education, older age, and higher levels of risk tolerance (Lown, 2011). Xiao et al. (2015) also found financial self-efficacy increases with age, which may account for the lack of decrease of financial confidence with age previously mentioned. In addition, financial self-efficacy has been associated with increased financial knowledge, behavior, and coping (Danes & Haberman, 2007). One positive coping behavior significantly associated with financial self-efficacy is seeking professional financial help (Lim et al., 2014), which also may serve as a protectant factor against FE. These findings provide strong evidence of the importance of financial self-efficacy as a protective factor against FE causing financial hardship. Although we know older adults do not necessarily lose financial confidence, high financial self-efficacy may lead them to be more open to seeking advice when faced with risky financial decisions.

### FE Vulnerability

Lichtenberg et al. (2020) tested the contextual variables related to financial decision making based on the conceptual framework from Lichtenberg et al. (2015), which was developed using a concept mapping approach. The study sought to identify how well contextual subscale questions differentiated those who had been victims of FE from those who had not. The study also investigated whether the contextual items that differentiated FE victims from nonvictims coalesced in a way that created a new, internally consistent scale: the Financial Exploitation Vulnerability Scale (FEVS). Using a community-based sample of 242 participants (78 of whom had confirmed FE, 40 of whom were also used in the current study), 17 contextual items formed the basis of an internally consistent scale that significantly differentiated the exploited from the nonexploited group (AUC = 0.82). Although there is some overlap of participants in the original FEVS study and the current study, it should be noted the empirical questions being investigated are not the same within the studies. The original FEVS study did not utilize the conceptual framework developed for the current study and did not use any of the financial resource or stressor variables that are the focus of this study.

Lichtenberg et al. (2021) followed up with a study of the criterion validity of the FEVS. This study used a sample of 258 individuals, aged 60 and older, who completed the FEVS on the https://olderadultnestegg.com website. Their results indicated a significant association between self-reported memory loss and increased FEVS scores as well as especially heightened FEVS scores among those living alone and rating their memory as declining over the past year.

### Importance of Focusing on Older African Americans

The existing body of FE literature provides evidence that African American older adults are at an increased risk of being victimized. Beach et al. (2010) and Laumann et al. (2008) reported increased risk of FE for African American older adults using random population-based samples. Beach et al. (2010) examined racial differences in the prevalence of FE and psychological mistreatment. African Americans showed significantly higher rates of being financially exploited since turning 60 and in the past 6 months. The prevalence for FE since turning 60 among African American older adults was nearly three times higher than non-African American older adults. Lichtenberg et al. (2016) also reported higher rates of FE for African American older adults in their community sample. Nevertheless, there continues to be a need to better understand FE causing financial hardship for African American older adults. This study purposely heavily recruited African American participants to provide empirical literature to broaden the understanding of this population and FE.

### Study Purpose and Research Questions

This study will examine the associations described in our proposed conceptual framework to better understand older adults’ vulnerability to FE causing financial hardship. First, we will examine the relationships between sociodemographic measures, financial stressor and resource measures, and FE causing financial hardship. Next, we will investigate the relationships of sociodemographic measures with financial stressors and resource measures. We will investigate which measures are significantly related to FE causing financial hardship. Finally, we will investigate the criterion-related validity of the FEVS and financial resources and stressors.

H1: In our bivariate analyses, individuals with a history of FE causing financial hardship will demonstrate significantly lower financial literacy and financial self-efficacy, higher financial hassles and FEVS scores, and lower levels of education.

H2: Level of education will be significantly correlated with financial stress and resource measures, and financial stress and resource measures will be significantly related to one another.

H3: The FEVS will be significantly related to FE causing financial hardship even when controlling for sociodemographic and financial stressor and resource measures.

H4: Financial self-efficacy (the financial resource measure) and financial hassles (the measure of financial stress) will be significantly associated with the FEVS independent of other measures.

## Method

### Procedures

Participants were selected from the Successful Aging through Financial Empowerment (SAFE; Lichtenberg et al., 2019) program and a community-based sample of a validation study for a financial decision-making scale (Lichtenberg et al., 2018). One hundred and forty-two community-dwelling older adults were included. Participants were recruited through referrals from local senior agencies, professionals, flyers, or participation in community education programs. Flyers were distributed at community education seminars on FE and community resource fairs targeting older adults. Recruitment of all participants took place in the same community over the same time period. The study was approved by the University’s Human Subjects Internal Review Board, and each participant signed an informed consent document before any assessments to allow their data to be used in this study.

The inclusion criteria were as follows for the SAFE sample: age 55 or older, a victim of FE causing financial hardship (e.g., scam, identity theft), living independently in the community, and able to read on at least a basic level. The inclusion criteria for the community-based volunteer sample were the same, with the exception of having experienced FE causing financial hardship.

### Participants

SAFE participants were referred by area professionals who work with older adults and/or by self-referral after attending a SAFE community education program and reporting having experienced FE. This report was further validated through bank records or online credit reports. The financial coach and SAFE clients gathered the proper financial records and reviewed them to locate discrepancies, such as fraudulent charges and unfamiliar accounts. If FE was verified through this process, the financial coach and SAFE client worked together to resolve any negative financial outcomes resulting from the FE (for a complete description see Lichtenberg et al., 2019).

In addition, community comparison group participants were asked a series of questions about financial decision making, during the Lichtenberg Financial Decision Rating Scale administration, including “Have you ever lost money due to a financial scam, exploitation, or identity theft?” Inclusion criteria for the community comparison sample were that participants did not experience FE after age 60 (Lichtenberg et al., 2017). Assessments were administered to the SAFE group by the financial coach, while the assessments of the community-based volunteer group were completed by a trained member of the research team. Assessments were conducted in the participant’s home, a community library, or our research office.

### Measures

#### Financial literacy

Three questions were used to determine the participants’ level of financial literacy. The questions included in this measure first appeared in the 2004 HRS. This three-question scale was designed to gauge the “knowledge of basic financial investment concepts, such as inflation, risk diversification and the capacity to do calculations related to interest rates” (Lusardi, 2012, p. 25). The total score range is 0–3, higher scores indicate higher levels of financial literacy.

#### Financial hassles

The Financial Hassles Scale was derived from the 117-item daily hassles scale. Twenty money-related items were used to gauge participants’ experiences with financial stressors. The cumulative severity measure, the sum of the 3-point severity ratings of 1, 2, or 3 meaning “somewhat,” “moderately,” or “extremely” was used to score the shortened scale (Kanner, 1981). The total score range for the Financial Hassles Scale is 0–60. Higher scores suggest greater levels of financial stress. The Financial Hassles Scale demonstrated good internal consistency (Cronbach’s alpha = 0.88).

#### Financial Self-Efficacy Scale

The Financial Self-Efficacy Scale (FSES) was used to assess participants’ perspectives on their ability to handle financial situations. The scale measures specific financial behaviors to assess an individual’s ability to deal with financial situations without being overwhelmed (Lown, 2011). The FSES consists of six questions rated on a 4-point Likert scale (1 = exactly true to 4 = not at all true). The internal consistency for this scale was good (Cronbach’s alpha = 0.75). Higher scores indicated higher levels of financial self-efficacy.

#### Financial Exploitation Vulnerability Scale

Participants in the study completed the 17-item FEVS (Lichtenberg et al., 2020). These self-report items ask about the context in which an older adult is making a financial decision. This context includes their financial circumstances (e.g., “How often do your monthly expenses exceed your regular monthly income?”) and the impact of their finances on their psychosocial health (e.g., “Has your relationship with a family member or friend become strained due to finances?” and “How often do you worry about financial decisions you have recently made?”). The 17 items on the FEVS have a risk score that ranges from 0 to 2 points or 0 to 3 points, depending on the number of response options. The total score range is 0–46, with higher scores relating to a higher risk of FE. The scale’s internal consistency was in the good range (Cronbach’s alpha = 0.76).

### Statistical Analysis

IBM SPSS Statistics 26 was used to analyze the data. Baseline data on SAFE and comparison group participants were used to complete the analysis. To test Hypothesis 1, bivariate analyses (*t*-tests and chi-squares) were performed to compare the FE resulting in financial hardship group and the community volunteer group on sociodemographic, financial stressor, financial resource, and FE vulnerability measures. To examine Hypothesis 2, a correlation matrix was performed to assess the strength and direction of the relationships among all sociodemographic factors, as well as the financial stressor and resource measures. Logistic regression was completed to test Hypothesis 3 that the FEVS measure would be significantly associated with FE even when accounting for other measures. Finally, a multiple regression approach was used to test whether financial resource and stressor measures were significantly associated with FEVS scores.

## Results

### Sample Characteristics

The sample consisted of 142 community-dwelling participants. Sixty-two of the participants (43.6%) were victims of FE causing such financial hardship that they sought SAFE services. All of the cases of FE causing financial hardship were substantiated through a review of their financial records by the SAFE director. The overall sample was predominately African American (83.1%), mostly female (78.9%), and largely unmarried (79.6%). The average age of participants was 69.55 years. On average, participants had 14.39 years of education (see Table 1 for more details).

**Table 1.** Sample Demographics and Financial Measures (*N* = 142)

Variable	FE history (*n* = 62)	No FE history (*n* = 80)	Overall sample (*N* = 142)	*t* or χ ^2^	Effect size
Marital status, *n* (%)				10.342**	π = −0.27
Married	5	24	29 (20.4%)		
Unmarried	57	56	113 (79.6%)		
Gender, *n* (%)				0.621	—
Male	15	15	30 (21.1%)		
Female	47	65	112 (78.9%)		
Race, *n* (%)				0.055	
African American	51	67	118 (83.1%)		
White	11	13	24 (16.9%)		
Age (years), *M* (*SD*)	69.32 (7.86)	69.72 (5.81)	69.55 (6.76)	0.351	—
Education (years), *M* (*SD*)	13.72 (2.13)	14.88 (2.46)	14.39 (2.39)	0.135**	*d* = 0.48
FSES (score range 4–24), *M* (*SD*)	14.52 (4.02)	16.21 (4.23)	15.48 (4.21)	2.397**	*d* = 0.40
Financial literacy (score range 0–3), *M* (*SD*)	1.98 (0.87)	2.29 (0.78)	2.16 (0.83)	2.181*	*d* = 0.37
Financial hassles (score range 0–60), *M* (*SD*)	13.66 (11.13)	7.72 (8.40)	10.28 (10.07)	−3.564**	*d* = −0.59
FEVS (score range 0–46), *M* (*SD*)	8.90 (4.45)	5.26 (4.09)	6.85 (4.61)	−4.972***	*d* = −0.79

*Note:* FE = financial exploitation; FSES = Financial Self-Efficacy Scale; FEVS = Financial Exploitation Vulnerability Scale.

*p < .05, **p < .01, ***p ≤ .001.

### Bivariate Associations of Demographic and Financial Measures With FE

*t*-Tests and chi-square analyses were used to determine group differences in demographics and financial stressor and resource measures. In terms of demographics, the FE and comparison group had significant differences in marital status (χ ^2^(1) = 10.34, *p* < .01) and educational attainment (*t* (139) = 0.135, *p* < .01). Those in the FE group were more likely to be unmarried and had completed fewer years of education. The effect size for education was moderate (*d* = 0.48). The FE group had significantly lower FSES scores (*t* (139) = 2.40, *p* < .01) and financial literacy scores (*t* (139) = 2.18, *p* < .05), while financial hassles (*t* (139) = −3.56, *p* < .01) and FEVS scores (*t* (139) = −4.97, *p* ≤ .000) were significantly higher for the FE group. The strongest bivariate relationship between the measures and FE status was the FEVS score. The effect size for FEVS was moderate to strong (*d* = 0.79; see Table 1 for more details). The results provided support for Hypothesis 1.

Demographic and financial stressor and resource measures were entered into a correlation matrix to gauge the direction and strength of their relationships. Marital status, race, gender, education, FSES, financial literacy, financial hassles, and FEVS were entered into the correlation matrix. A significant and negative relationship was found between FSES and FEVS (*r* = −0.59, *p* < .01). A strong positive correlation was found between financial hassles and FEVS (*r* = 0.71, *p* < .01). The total years of education were significantly related to financial literacy, financial hassles, and the FEVS (see Table 2 for more details). Thus, the results provided support for Hypothesis 2.

**Table 2.** Correlations Between Demographics and Financial Measures (*N* = 142)

Variable	Age	Marital status^a^	Race^b^	Gender^c^	Education	FSES	Financial literacy	Financial hassles
Marital status^a^	0.037							
Race^b^	−0.133	0.191*						
Gender^c^	−0.068	0.209*	0.135					
Education	−0.066	0.222**	−0.004	−0.021				
FSES	0.104	0.192*	−0.007	0.085	0.166			
Financial literacy	0.034	0.201*	0.074	0.300**	0.314**	0.136		
Financial hassles	−0.110	−0.267*	−0.009	−0.039	−0.228**	−0.538**	−0.125	
FEVS	−0.173*	0.303**	0.056	0.009	−0.192*	−0.589**	−0.011	0.708**

*Note:* FSES = Financial Self-Efficacy Scale; FEVS = Financial Exploitation Vulnerability Scale.

^a^Single is the reference group.

^b^African American is the reference group.

^c^Female is the reference group.

*p < .05, **p < .01.

### Multivariate Analysis

A logistic regression model was created to assess Hypothesis 3. The logistic regression model was statistically significant (χ ^2^ = 35.674, *p* < .001) and explained 32.5% (Nagelkerke *R*^2^) of the variance in FE victimization within the sample. This model correctly classified 79.1% of cases. Financial literacy and FEVS scores held significant associations with FE which causes financial hardship, while none of the sociodemographic or other financial stress and resource measures were significantly related to FE. Hypothesis 3 was partially supported in that the FEVS was significantly associated with the FE group, but was not the only financial stress and resource measure to be significantly related to FE. Financial literacy had a significant relationship with FE in the logistic regression (see Table 3 for more details).

**Table 3.** Logistic Regression Demographic, Financial Measures, and FEVS on Scam and ID Theft outcome (*N* = 142)

Variable	*Β*	*SE*	Wald	df	Sig.	Exp (*B*)
Age	0.007	0.031	0.053	1	.817	1.007
Marital status^a^	−1.070	0.619	2.986	1	.084	0.343
Race^b^	−0.218	0.590	0.137	1	.712	0.804
Gender^c^	1.140	0.546	4.365	1	.037	1.004
Education	−0.113	0.097	1.360	1	.243	0.893
FSES	−0.070	0.065	1.162	1	.281	1.073
Financial literacy	−0.596	0.284	4.396	1	.036*	0.551
Financial hassles	0.027	0.031	0.734	1	.392	1.027
FEVS	0.159	0.071	5.024	1	.025*	1.172
Constant	−0.049	3.324	0.000	1	.988	0.953

*Note*: FSES = Financial Self-Efficacy Scale; FEVS = Financial Exploitation Vulnerability Scale.

^a^Single is the reference group.

^b^African American is the reference group.

^c^Female is the reference group.

*p < .05.

Results from the linear regression to test Hypothesis 4 are given in Table 4. The multiple regression model was statistically significant, *F*(9, 128) = 20.344, *p* ≤ .000, adj. *R*^2^ = 0.58. Financial hassles and FSES were significantly associated with FEVS. Financial hassles had a significant positive association with FEVS scores (*B* = 0.233, *p *≤ .000), while FSES had a significant negative association (*B* = −0.278, *p* ≤ .000) with FEVS. No significant association was found between financial literacy and FEVS.

**Table 4.** Multiple Regression Demographic and Financial Health Measures on FEVS (*N* = 142)

Variable	*Β*	*SE*	Beta	*t*	Sig.
Age	−0.076	0.041	−0.112	−1.846	.067
Marital status^a^	−1.495	0.698	−0.138	−2.142	.034
Race^b^	0.639	0.718	−0.054	−0.890	.375
Gender^c^	0.400	0.685	0.037	0.584	.560
Education	−0.066	0.121	−0.036	−0.551	.583
FSES	−0.278	0.076	−0.256	−3.649	.000***
Financial literacy	0.556	0.357	0.102	1.559	.122
Financial hassles	0.233	0.032	0.522	7.235	.000***
Constant	15.145	4.011		3.776	.000***

*Note:* FSES = Financial Self-Efficacy Scale; FEVS = Financial Exploitation Vulnerability Scale.

^a^Single is the reference group.

^b^African American is the reference group.

^c^Female is the reference group.

***p ≤ .001.

## Discussion

The major finding of this study is that it tested the relationships between FE causing financial hardship and individual characteristics using a conceptual model that included both sociodemographic and financial stress and resource measures. Univariate measures demonstrated the association of both sociodemographic and financial stress and resource measures with FE. While sociodemographic and financial stress and resource measures had significant correlations with one another, the logistic regression model found both financial literacy and FEVS were the only measures that held significant relationships with FE causing financial hardship. It was expected that FEVS would be a robust predictor of FE causing financial hardship and that the variance in the outcome attributed to financial self-efficacy, financial literacy, and financial hassles would not be significant when FEVS was also in the regression. In fact, the study results indicated that the FEVS and financial literacy measure both had a unique and significant association with the FE measure. Interestingly, FEVS and financial literacy were not significantly related to one another in the analysis exploring the criterion validity of the FEVS.

As expected, financial self-efficacy and financial hassles were significantly associated with FEVS. FEVS is a contextual financial decision-making scale that encompasses financial strain, self-efficacy, financial behaviors and psychological vulnerability with respect to finances, and conflicts and relationship strain related to finances. The initial validation study of the scale proved it to be useful in differentiating who had been a victim of FE causing financial hardship and who had not. Lichtenberg et al. (2021) provided evidence of the FEVS criterion validity by examining how memory loss and living alone were related to FEVS scores. The findings from this study further support the criterion validity of the scale as it relates to an individual’s perception of their financial abilities and stressors. Financial literacy, however, corresponds less to one’s perceptions of finances and more to the understanding of financial concepts and the ability to calculate accurate financial word problems. Financial literacy is akin to the intellectual factors in our financial decision-making conceptual framework (see Lichtenberg et al., 2015) in that it involves an understanding and appreciation of applied financial concepts and problems.

We use the term FE causing financial hardship intentionally in order to encourage a greater specificity in describing those older persons who experienced FE. The impact of identity theft or a scam that results in no financial hardship is vastly different than when it undermines one’s financial stability (e.g., alleged debt and ruined credit). Those who experience financial hardship after FE also are unsure of how to correct their ruined credit and dispute the alleged debt, and often ineffective when trying to do so. Hall et al. (2021) provided preliminary evidence that those who experienced FE with financial hardship had lower executive functioning, more mental health problems, and more problems with Instrumental Activity of Daily Livings (IADLs) at baseline than a group matched on age and education. At the 6-month follow-up after individual financial assistance services were given, the FE group had significantly less anxiety than at baseline and had trends toward improved executive functioning and IADLs. Greater specificity in FE terms can help increase our understanding of the impact of FE, as much as falls and injurious falls have been differentiated in the gerontology literature (Cai et al., 2021).

In addition to the important contribution of understanding financial stressors and resources and their relationship with FE causing financial hardship, the current study fills an important gap in the literature on older African Americans and FE. It is important to note that while previous studies focusing on older African Americans investigated risk and prevalence estimates of FE in general (Beach et al., 2010; Lauman et al., 2008; Lichtenberg et al., 2016), the current study actually examines the relationships between FE causing financial hardship and financial attitudes, stressors, and skills within this population. It provides a framework to understand how financial stressors and resources affect the FE causing financial hardship experienced by this population.

### Limitations

This study is not without its shortcomings. First, it is a cross-sectional design, so we cannot test the predictive ability of our model. Second, the non-FE group is a convenience sample that limits the generalizability of the findings. All of our findings are associations. The findings, however, are important, because it is one of the few studies to examine associations of financial stressors and resources to FE with financial hardship. It is also one of the few to do so in a sample of predominately older African American women.

In addition, the study is limited in its ability to investigate the full conceptual framework. Due to the study’s use of convenience sampling, the sample is small. To adhere to the suggested minimum number of observations per covariate, the factors (health and cognitive status) in the conceptual framework were not added into the manuscript’s regression models. Although this study focuses on the sociodemographic and financial measures and their associations with FE causing financial hardship, the association of the health and cognitive status measures included in the conceptual framework and FE has been validated through previous studies on the SAFE program (Hall et al., 2021; Lichtenberg et al., 2019). This leads the study’s authors to believe the inclusion of these measures as covariates would result in positive associations and produce a model with more predictive power. Larger sample sizes in future studies will give researchers the ability to address this limitation.

Another potential weakness of the current study is the threat of social desirability bias created through having the financial coach collect the baseline data of the SAFE participants. In an attempt to control for this type of bias, all interviewers were trained in the same fashion and the community sample was interviewed by a separate member of the research team. It is also important to note that all intake assessment data were collected by the financial coach before the client received any services to address their financial hardship in an attempt to minimize social desirability bias. However, future studies’ capacity to utilize third-party assessment administrators, not financial coaches, as data collectors would possibly minimize this bias threat.

A final limitation noted by the authors is the study’s use of a sample of predominately African American older adults. Due to the oversampling of older African Americans, there may be some issues with generalizing the results to non-African Americans. However, it should be noted the instruments used to collect the financial stressor and resource measures were validated using non-Black samples. These aspects of the literatures are rooted in non-Black samples. Therefore, it is expected that there would be no differences when these relationships were investigated using samples not heavily populated with African Americans.

### Implications for Practice

The results from this study lead to three practice implications that could assist individuals in providing services to older adults. The first implication is that the conceptual framework supported here could be a guiding factor in understanding the relationship between the proposed financial resources and stressors to FE causing financial hardship. Individuals providing services to older adults now have a model to look to in order to understand this relationship. In addition, the findings provide some idea of the types of services that could be offered to protect older adults from becoming victims of FE causing financial hardship. Financial literacy and education offerings could increase the protective factors of financial self-efficacy and decrease the number of financial stressors older adults are faced with by providing more specific knowledge around finances and financial situations commonly experienced by the population. The third and final implication for practice is the use of the FEVS to determine if a client is at risk of FE causing financial hardship. As previously mentioned, the initial validation study of the scale proved it to be useful in differentiating who had been a victim of FE causing financial hardship and who had not. This provides practitioners with a tool to assess older adults for risk that was not available before. The scale is short, 17 questions, available online (https://olderadultnestegg.com), and generates a score and risk scale that can be used to determine if further intervention action needs to be taken to protect a vulnerable older adult from being victimized.
